# Wekemo Bioincloud: A user‐friendly platform for meta‐omics data analyses

**DOI:** 10.1002/imt2.175

**Published:** 2024-02-13

**Authors:** Yunyun Gao, Guoxing Zhang, Shunyao Jiang, Yong‐Xin Liu

**Affiliations:** ^1^ Shenzhen Branch, Guangdong Laboratory of Lingnan Modern Agriculture, Genome Analysis Laboratory of the Ministry of Agriculture and Rural Affairs, Agricultural Genomics Institute at Shenzhen Chinese Academy of Agricultural Sciences Shenzhen China; ^2^ Shenzhen Wekemo Technology Group Co., Ltd. Shenzhen China

**Keywords:** bioinformatics, meta‐omics, microbiome, user‐friendly platform, Wekemo Bioincloud

## Abstract

The increasing application of meta‐omics approaches to investigate the structure, function, and intercellular interactions of microbial communities has led to a surge in available data. However, this abundance of human and environmental microbiome data has exposed new scalability challenges for existing bioinformatics tools. In response, we introduce Wekemo Bioincloud—a specialized platform for ‐omics studies. This platform offers a comprehensive analysis solution, specifically designed to alleviate the challenges of tool selection for users in the face of expanding data sets. As of now, Wekemo Bioincloud has been regularly equipped with 22 workflows and 65 visualization tools, establishing itself as a user‐friendly and widely embraced platform for studying diverse data sets. Additionally, the platform enables the online modification of vector outputs, and the registration‐independent personalized dashboard system ensures privacy and traceability. Wekemo Bioincloud is freely available at https://www.bioincloud.tech/.

## Highlights

- Wekemo Bioincloud offers not just workflows for diverse meta‐omics data analysis but also a rich array of tools for effective data visualization.
- Enables modifying vector outputs online, achieving publication‐ready figures.
- Displays tools' popularity/usage trends, enhancing the platform's flexibility and user‐friendly experience.

## Graphical abstract

Wekemo Bioincloud is a specialized platform for meta‐omics studies that addresses scalability challenges in handling vast microbial community data with 22 workflows and 65 visualization tools. It provides comprehensive analysis, online vector output modification, and ensures privacy with a registration‐independent dashboard system.

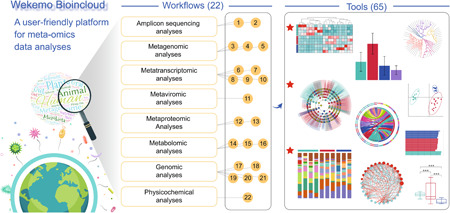

## INTRODUCTION

Recent development of meta‐omics approaches, spanning metagenomics, metatranscriptomics, metaproteomics, metaviromics, metabolomics, and physicochemical data, mark a transformative era in the comprehensive understanding of intricate biological systems [1, 2, 3]. The resulting multi‐omics data sets, encompassing the richness of microbial communities, present a profound need for robust bioinformatics tools that are both robust and user‐friendly [4], to unveil the profile of microbial communities and elucidate the nuanced interactions between environmental conditions and microbiome. Advancements in analytic platforms tailored for high‐throughput omics data further underscore the evolution in bioinformatics capabilities. Noteworthy examples include the application of QIIME 2 [5] and EasyAmplicon [6] for amplicon data analyses, Trimmomatic [7] or fastp [8] for stringent quality control, Kraken 2 [9] for precise taxonomic classification, HUMAnN3 pipeline [10] for comprehensive functional profiling, MultiPrime [11] for efficient minimal primer design, imageGP [12] for data visualization, and more, which collectively contribute to a more profound exploration of diverse omics data sets.

Conceptually, the standard omics data analysis workflow contains raw data processing, taxonomy annotation, functional profiling, and statistical analysis. Several workflows have been developed to streamline these processes [4, 13, 14, 15]. However, in diverse research contexts, personalized analysis approaches are crucial, emphasizing the necessity for customized analyses. Nowadays, various tools, pipelines, and online web services have been developed for ‐omics analyses. For instance, QIIME 2 [5] is a software primarily designed for amplicon sequencing analyses, which has expanded its capabilities to include metagenomic analyses. EasyAmplicon [6] is a pipeline specialized in amplicon sequencing analyses on the local server. MicrobiomeAnalyst [16] operates as a web server, mainly for amplicon sequencing analyses, metagenomic analyses, and metabolomic profiling. Notame [17] presents a dedicated workflow for metabolomic profiling. MetaProteomeAnalyzer [18] is a workflow for metaproteomic data analyses. Meanwhile, numerous innovative approaches have emerged for identifying reliable and stable biomarkers from ‐omics data [19, 20, 21, 22], and several research have diligently summarized and compared various R packages or software tools designed for ‐omics data [23, 24, 25, 26, 27, 28, 29, 30, 31]. However, the majority of these tools are oriented toward one or two specific types of ‐omics data analyses. Currently, integrative analysis across multiple ‐omics has become crucial for addressing scientific questions [1, 2]. Nevertheless, the diversity and complexity of analytical approaches mean that researchers not only need to install various tools or R packages for data analysis but also invest significant time in adapting to different tools or platforms. This highlights a considerable gap in the scientific community, emphasizing the need for an easily accessible web service specifically designed for the analysis and visualization of meta‐omics data [32].

Here, we present Wekemo Bioincloud, tailored for specific ‐omics studies, offering a comprehensive analysis solution that addresses the challenge of tool selection for users. The Wekemo Bioinclud platform securely stores users' raw sequencing data in the cloud and performs preprocessing tasks for various analysis tools in advance. Comprising two key modules, workflows and tools, the Wekemo Bioinclud platform satisfies a spectrum of requirements. Notably, this platform empowers users to oversee critical steps with dependencies in the workflow, facilitating systematic exploration of data and unveiling biological significance. This platform is openly accessible at https://www.bioincloud.tech/. For an in‐depth understanding of the platform, its usage, and result interpretation, comprehensive details can be found on the website.

## RESULT

### Overview of Wekemo Bioincloud

**Figure 1:**
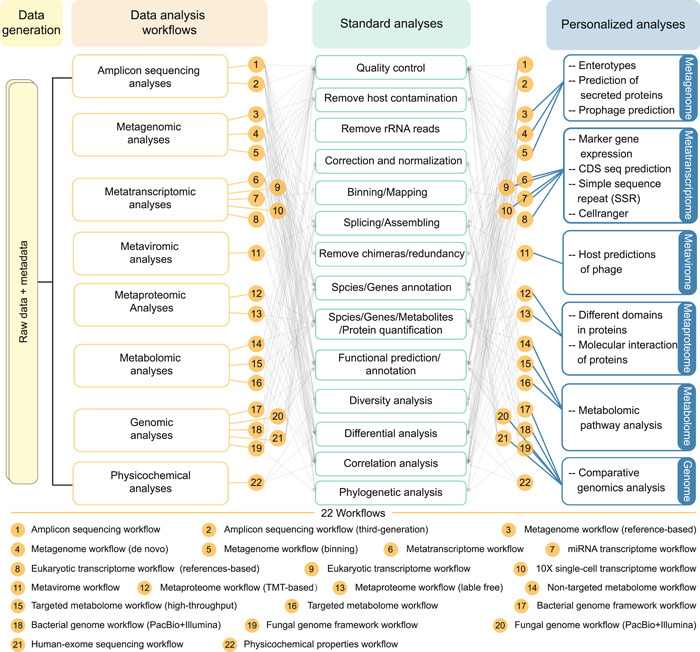
The framework of 22 analysis workflows for Wekemo Bioincloud. The platform now provides diverse workflows for meta‐omics data and accommodates both standardized and personalized analyses for various research. CDS, coding sequences; mRNA, messenger RNA; rRNA, ribosomal RNA; TMT, tandem mass tag.

The Wekemo Bioincloud comprises two main components: the workflows and the tools. In the workflow module, users can analyze omics data step by step, generating reports that detail the software used and their respective parameters for each analysis (Figure 1). The tool module allows users to easily showcase their data, referencing our demo files. The platform is designed to be as convenient as possible for researchers to access analyses, which can modify all groups with one click in all analyses, and also run all analyses with one click. Notably, we offer more than just scalable vector graphics (SVG) editors for refining output images; users can also set up email reminders for each step, saving them valuable time. Additionally, we provide instructional videos covering tool usage, various workflows, and result analyses, enhancing user comprehension of their data. Our registration‐independent personalized dashboard system ensures privacy, traceability, and collaboration. There are two ways for researchers to use our platform. First, they have the option to analyze their data by providing tables through our tool module, referring to our online demo table. Alternatively, they can choose to submit their raw data to us. In return, we furnish them with standard analysis tables, then users have the flexibility to conduct personalized analyses either through our workflows or by using our tools online. All raw data and result reports for workflow analyses will be retained for 2 years, offering ample time for users to mine the data in depth, while the data used for tools will be deleted every day.

After a thorough examination of all publications referencing Wekemo Bioincloud on Google Scholar until December 6, 2023, we observed that 157 publications have cited our platform. Following the removal of 17 publications due to unknown or repeated entries, a total of 140 distinct publications were identified. Notably, 42.14% of these publications opted for our workflows module, while the remaining 57.86% chose the tools module for visualizing their results. Common techniques employed for visualization include correlation tests, orthogonal partial least‐squares discriminant analysis (OPLS‐DA), principal coordinate analysis (PCoA), and linear discriminant analysis effect size (LEfSe) analysis (see Table S1).

### Cloud workflows for meta‐omics data

The workflows module has currently been updated with 22 data analysis workflows, encompassing one‐step analyses of various types of data, including metagenome, metatranscriptome, metaproteome, metavirome, metabolome, genome, and physicochemical data (Figure 1). Each workflow comes with a comprehensive demo report, example processes, and interpretation of results, facilitating a quick start for new users. For routine ‐omics analysis, users only need to prepare raw sequencing data and metadata information by referring to our demo pipeline. Faced with plenty of meta‐omics analysis software, our workflows also include various software options. Users can easily choose different analysis algorithms/software based on the specific characteristics of their data, and all processing methods will be showcased in the output report.

Furthermore, we also provide some flexible choices for users to gain their personalized analysis. For example, the metagenomic pipeline can categorize the enterotypes of microbiome samples [33], and predict the prophage or secreted proteins of metagenomic binning. For profiling the transcriptional activity of individual cells, the 10× single‐cell transcriptome workflow empowers CellRanger (http://10xgenomics.com) to handle output results, incorporating processes such as alignment, quantification, clustering, and gene expression analysis. In addition, we have integrated different technologies for analyzing bacterial and fungal genomes using only Illumina short‐read sequencing, or Illumina short‐read sequencing with PacBio long‐read sequencing, facilitating the systematic exploration of data and unveiling biological significance.

### Graphical tools for different purposes

**Figure 2:**
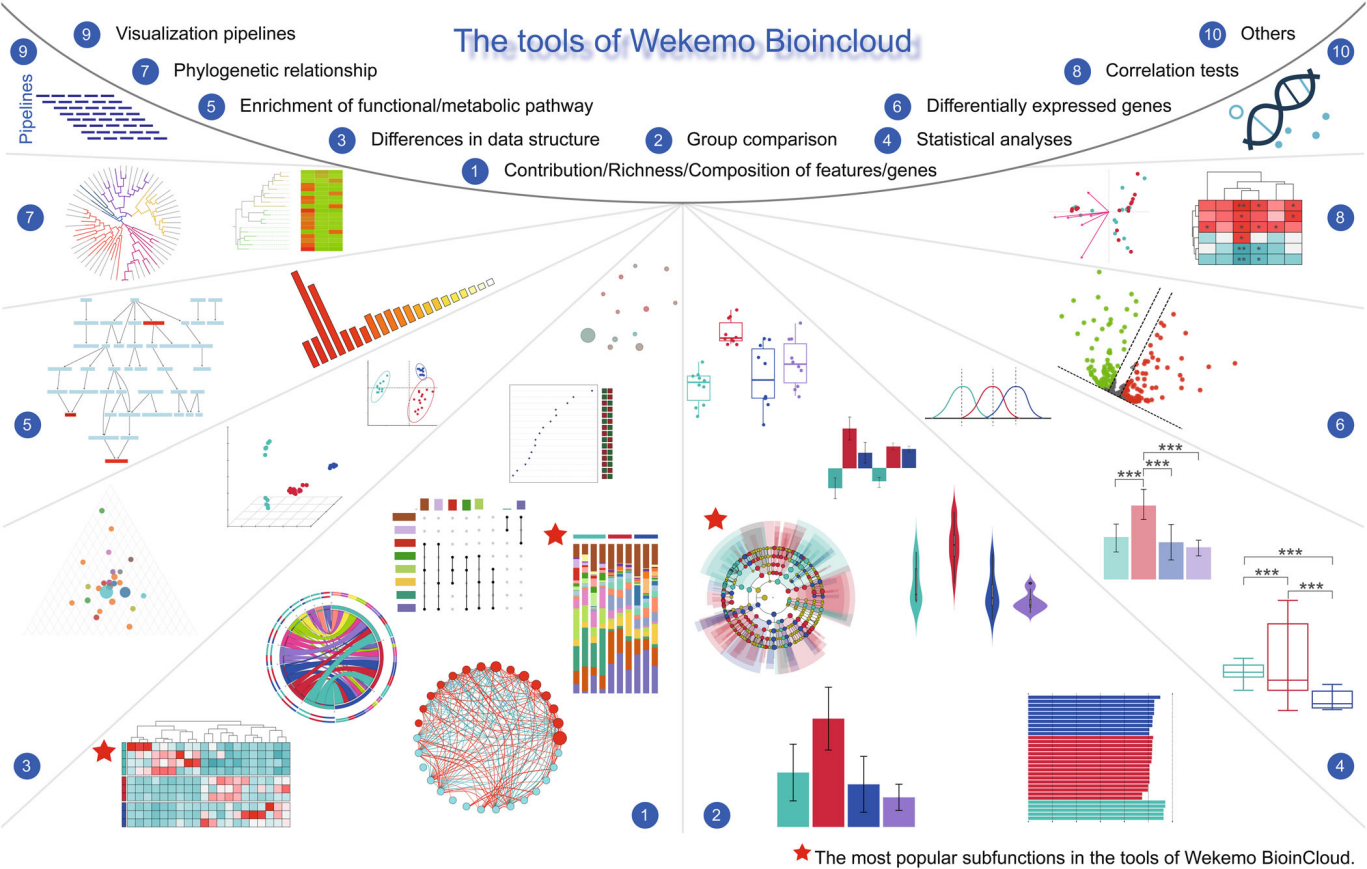
Example outputs generated by Wekemo Bioincloud Tools. The tools now contain 65 subfunctions, serving various purposes, including (1) the presentation of contribution, richness, composition of features or genes; (2) group comparison; (3) analysis of differences in data structure; (4) statistical analyses, such as analysis of variance tests, Kruskal–Wallis tests, and so forth; (5) enrichment of functional or metabolic pathways; (6) identification of differentially expressed genes; (7) construction of phylogenetic relationship; (8) correlation tests; (9) visualization pipelines, such as amplicon sequencing pipeline, metagenome taxonomy annotation pipeline, and so forth; (10) others, such as primer design, and so forth.

To facilitate the intuitive presentation of scientific discoveries, the Bioincloud platform offers a diverse array of tools for visualizing, analyzing, and comparing the ‐omics data (Figure 2). Currently, it has launched 65 subfunctions, covering the analyses of (1) contribution, richness, composition of features or genes, (2) group comparison, (3) differences in data structure, (4) statistical analyses, (5) functional or metabolic pathways, (6) differentially expressed genes, (7) phylogenetic relationship, (8) correlation tests, (9) visualization pipelines, and (10) others. All subfunctions with popularity and difficulty scores help users gauge the usage frequency and complexity of each tool. The grouped cluster heatmap, LEfSe plots, and grouped percentage stacked bar plots are currently the three most popular tools, with usage counts reaching 36,098, 34,261, and 33,341, respectively, by December 10, 2023.

The tools module covers the majority of daily needs for ‐omics researchers through its 65 visualization and analysis functions, involving scatter plots, bar plots, bubble plots, violin plots, network plots, ternary plots, volcano plots, petal plots, heatmaps, pathway diagrams, and more (Figure 2). Additionally, basic significance tests such as analysis of variance (ANOVA), Kruskal–Wallis, and Dunn tests are available, along with common molecular tools like 16S ribosomal RNA (rRNA) gene blast and primer design. Moreover, it provides features such as the conversion of SVG to various formats, including PDF, JPG, PNG, and others. All detailed explanations and demo data sets are available on the website to address any potential user misunderstandings.

### Case 1: Metagenomic data analysis workflows

**Figure 3:**
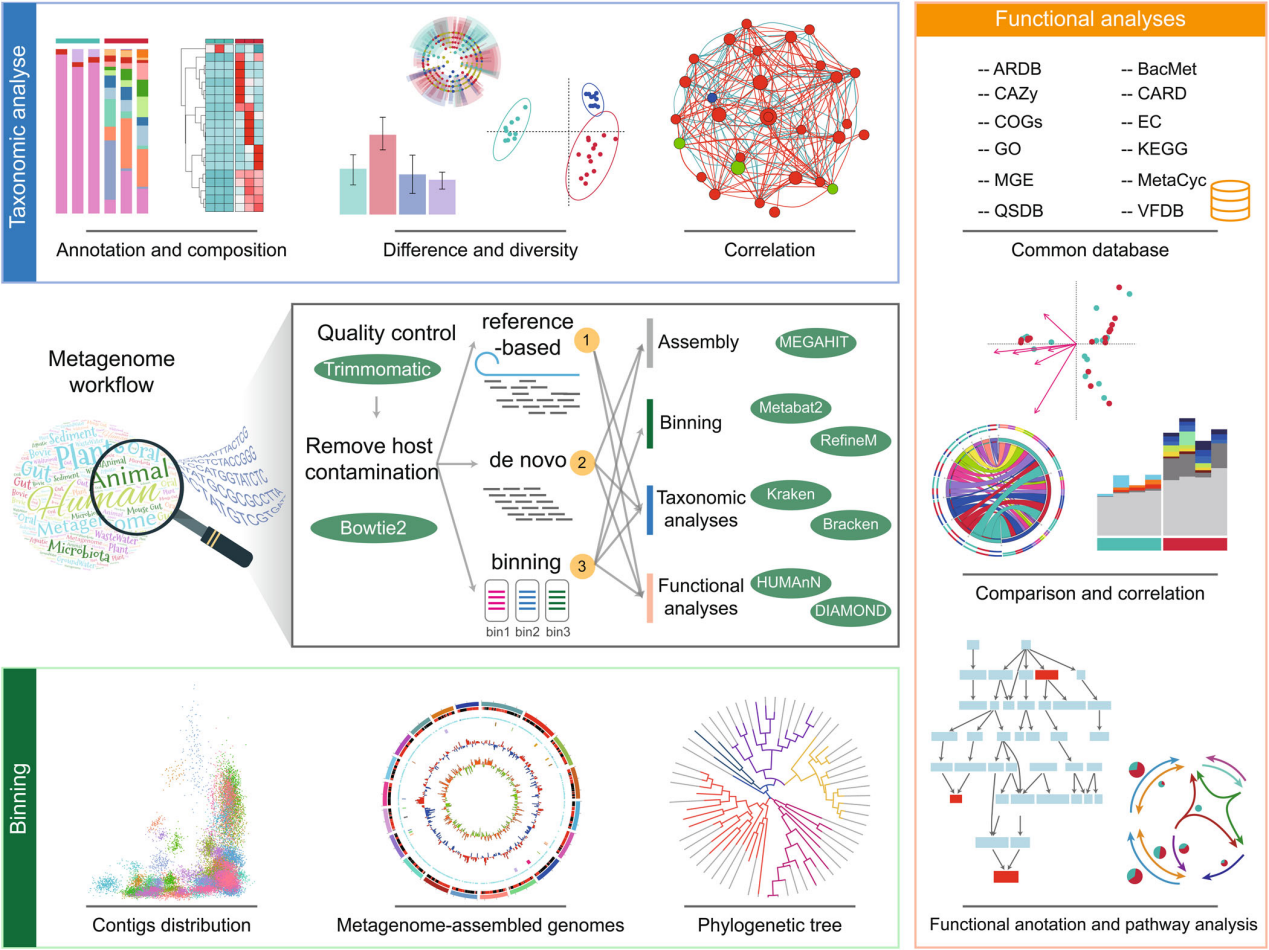
Metagenomic data analyses using Wekemo Bioincloud platform. The clean data could be analyzed with three different workflows, the reference‐based, the de novo, and the binning. The main software and visualization during assembly, binning, taxonomic analyses, and functional analyses were presented, making it suitable for various purposes or scenarios. The functional analyses include 12 diversity databases. ARDB, Antibiotic Resistance Genes Database; BacMet, Antibacterial Biocide and Metal Resistance Genes; CARD, Comprehensive Antibiotic Resistance Database; CAZy, Carbohydrate‐Active EnZymes; COGs, Clusters of Orthologous Groups of proteins; EC, Enzyme Commission; GO, Gene Ontology; KEGG, Kyoto Encyclopedia of Genes and Genomes; MetaCyc, Metabolic Pathway; MGE, Mobile Genetic Element; QS, Quorum Sensing; VFDB, Virulence Factors Database.

The platform offers three workflows for analyzing metagenomic data, the reference‐based workflow, the de novo workflow, and the binning workflow (Figure 3). All raw reads are processed using KneadData (https://github.com/biobakery/kneaddata) to obtain clean reads, with Trimmomatic [7] used for the trimming of adapter sequences and low‐quality reads, and Bowtie employed for the removal of host genome contamination. Following this, all clean reads undergo further processing for various purposes.

Notably, we leverage 12 commonly used bioinformatics databases in metagenomic analyses to predict the functions.

- ARDB [34]: Antibiotic Resistance Genes Database is used to track antibiotic resistance genes.
- BacMet [35]: Antibacterial Biocide and Metal Resistance Genes Database is used to confer the resistance to metals or antibacterial biocides.
- CAZy [36]: Carbohydrate‐Active EnZyme is used to describe enzyme families responsible for cleaving or building complex carbohydrates.
- CARD [37]: Comprehensive Antibiotic Resistance Database is employed to identify antibiotic resistance and virulence factors.
- COGs [38]: Clusters of Orthologous Groups of proteins are attempted on a phylogenetic classification of the proteins.
- EC [39]: Enzyme Commission, the numbers represent enzymes and enzyme genes. Evolutionary gene genealogy.
- GO [40]: Gene Ontology, annotations report connections between gene products and the biological types.
- KEGG [41]: Kyoto Encyclopedia of Genes and Genomes is utilized to identify functions within the biological system.
- MGE [42]: Mobile Genetic Elements is used to carry various kinds of genes endowing their hosts with resistance to antibiotics and/or metals, pathogenicity, symbiosis, and metabolism of new substrates.
- MetaCyc [43]: It contains pathways involved in both primary and secondary metabolism.
- QSDB [44]: Quorum Sensing Database, a phenomenon in which the accumulation of signaling molecules allows a single cell to perceive the number of bacteria, enabling coordinated responses and behaviors among bacterial cells.
- VFDB [45]: Virulence Factors Database is accessed to get bacterial virulence factors.

The reference‐based workflow involves mapping raw reads to different databases, including gene, nucleotide, or protein sequences. In this approach, Kraken [9] is utilized for taxonomic analyses with clean reads, and Bracken [46] is employed for estimating the species‐ or genus‐level abundance. To present the annotation or composition of species, bar plots, heatmaps, and Venn plots can be used. Comparisons between groups or samples can be demonstrated using ANOVA [47], DESeq2 [48], Kruskal–Wallis [49], and LEfSe [50]. The diversity of groups can be visualized through Bray–Curtis nonmetric multidimensional scaling, Bray–Curtis PCoA, and α‐diversity analyses. Correlations between groups or different factors can be explored using heatmaps, networks, and redundancy analysis/canonical correspondence analysis. Furthermore, clean reads are assigned to microbial metabolic pathways and functions using HUMAnN and the UniRef90 diamond annotated full reference database. CARD, COG, KEGG, MetaCyc, GO, EC, and CAZy are employed for function analyses. Then, the annotation or composition, comparison, or correlation of functional analyses can be shown like taxonomic analyses. In addition, using DiTing [51] enables the analysis of elemental cycles (carbon, nitrogen, phosphorus, sulfur) and the creation of cycle pathway diagrams. Besides, users can also select differential genes, comparing their differences or functional pathways.

The de novo workflow revolves around generating assembled contigs without relying on existing reference sequences, which allows for the discovery of more poorly described taxonomic groups. In this process, the classification and analyses of taxonomy follow a similar approach to the reference‐based workflow, which was mentioned earlier. However, the function analyses are based on the assembled contigs, and assembly is conducted using MEGAHIT [52], followed by gene prediction using Prodigal [53]. The CARD, COG, KEGG, GO, CAZy, MEG, ARDB, BacMet, and VFDB databases are used for functional annotation. Gene counting, which refers to the number of genes within a particular functional category, can also be calculated.

The binning workflow takes advantage of multiple features, such as the co‐abundance and coverage of contigs across samples, as well as the grouping of contigs based on similar Kmer frequencies and GC content. In this pipeline, the MEGAHIT [52] is applied to assemble clean reads and yields contigs. Then, MetaBAT [54] is employed to bin the contigs, RefineM [55] is used to eliminate contigs for removing the high contamination contigs, CheckM [56] is utilized to evaluate the completion and contamination of each bin, and dRep [57] is used to obtain a nonredundant bin. Then, the analyses and visualization for composition and function of bins can be shown as described in reference‐based workflow. The CAZy, COG, GO, KEGG, VFDB, MGE, and CARD databases are used for functional annotation. In addition, the metagenomic assembled genes of bins can be created to display the information about chromosome orientation GC content or GC skew of different contigs.

### Case 2: Metabolomics data analysis workflow

**Figure 4:**
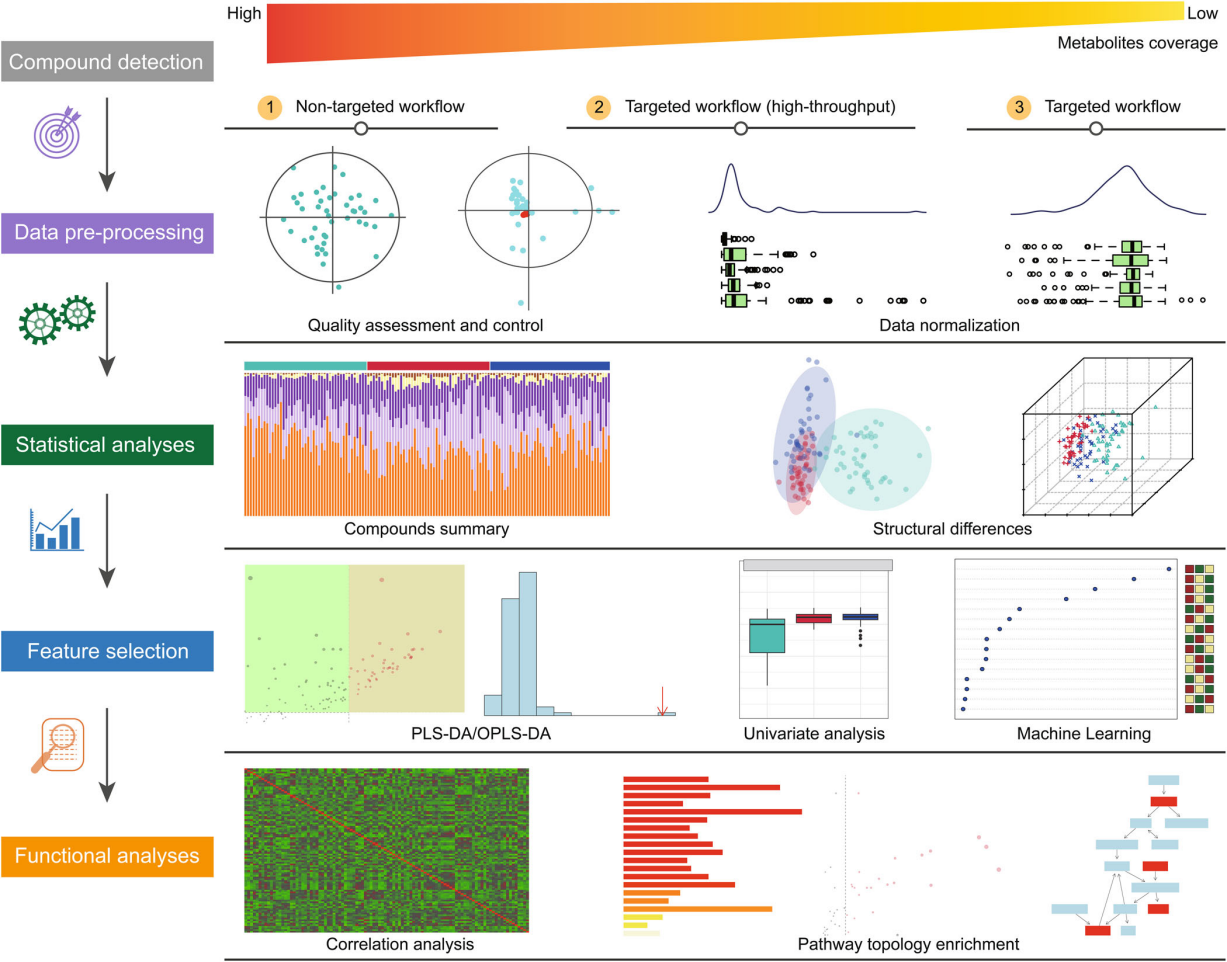
Metabolomic data analyses using the Wekemo Bioincloud platform. After employing three ways (nontargeted screening, targeted screening with high throughput and targeted screening) for compound detection, data preprocessing, statistical analyses, feature selection, and functional analyses are implemented in three workflows. Nontargeted screening provides the highest metabolite coverage, whereas targeted screening yields the lowest coverage. OPLS‐DA, orthogonal partial least‐squares discriminant analysis; PLS‐DA, partial least‐squares discriminant analysis.

Metabolomics can be broadly categorized into nontargeted and targeted metabolomics. Here, we offer three workflows, the nontargeted workflow, the targeted workflow, and the high‐throughput targeted workflow (Figure 4). Nontargeted metabolomics, characterized by its unbiased approach, facilitates a comprehensive analysis of the metabolites derived from the organisms, helping us to find some novel biomarkers. Targeted metabolomics employs standards, providing the absolute quantification of targeted metabolites, and it is reproducible. The high‐throughput targeted metabolomics allows the rapid and efficient analysis of a large number of metabolites in a sample, contributing to a more thorough understanding of the targeted metabolic profile. However, only targeted metabolomics can achieve absolute quantification of metabolites, the nontargeted and the high‐throughput targeted metabolomics are generally considered quantitative rather than absolute. The data analyses of the three different methods to gain metabolomics are mostly similar, containing compound detection, data preprocessing, statistical analyses, feature selection, and functional analyses.

MetaboAnalystR [26] is used to perform the nontargeted and targeted metabolomics data for potential detection of metabolite compounds in our platform.

- For data preprocessing, we implement quality assessment and quality control to detect the outliers, and to remove metabolites or samples beyond threefold standard error. Then, users have the option to decide whether to perform data normalization, eliminating potential systematic biases during sample collection or metabolite detection.
- After that, all metabolites are compared with the KEGG database br08001 [41] to determine the percentage content of each biological role. Standard statistical analyses, including compound summaries and the identification of structural differences, are then conducted.
- For feature selection, we offer partial least‐squares discriminant analysis (PLS‐DA) [58] or OPLS‐DA [59] to underlying metabolite patterns discriminating between sample groups. Then, the univariate analysis and machine learning techniques, such as random forest and support vector machine, are also employed for the selection of differential metabolites.
- For functional analyses, we offer the correlation analyses, over‐representation analysis (ORA) of pathways [60], topology analysis of pathways, and metabolic pathway maps, for providing intuitive insights into the relationships, functional patterns, and topological structure of metabolites within pathways.

Additionally, we apply maSigPro [61] package to enhance the analysis of metabolism time‐series data for nontargeted workflow and targeted workflow. This is achieved by implementing a generalized linear model, allowing users to identify significant regression relationships between various elements (such as genes, metabolites, or features) and temporal factors (like time, time squared, or specified groups). As part of our efforts, we have constructed a comprehensive high‐throughput targeted metabolism database, with more than 2500 plant metabolites and approximately 1800 animal metabolites.

## DISCUSSION

Wekemo Bioincloud platform offers a robust and user‐friendly service, contributing to global collaborative initiatives in the field of multi‐omics research. In comparison to existing online servers for ‐omics data analyses, such as MicrobiomeAnalyst [16], which focused mainly on metagenomic analyses, MetaboAnalystR [26] was specifically tailored for metabolomic analyses, and GeNets [62] was dedicated to genomic analyses. Our platform stands out by providing 22 workflows (Figure 1), encompassing the analysis of amplicon sequencing data, metagenomic data, metatranscriptomic data, metaviromic data, metaproteomic data, genomic data, and physicochemical data. This extensive coverage facilitates the integrated analyses of multiple ‐omics data types, offering researchers a comprehensive and user‐friendly solution.

sMeanwhile, Wekemo Bioincloud platform also provides some personalized analyses tailored to researchers, aligning with their diverse research scopes. For instance, we provide comprehensive bioinformatics databases (ARDB, BacMet, CAZy, CARD, COGs, EC, GO, KEGG, MGE, MetaCyc, QS, VFDB) for in‐depth functional analyses of metagenomic data (Figure 3). The platform supports comparison of enterotypes in different groups, and the prediction secreted of proteins or prophage in samples (Figure 1). To our knowledge, while Majorbio Cloud is also a bioinformatic platform for multi‐omics analyses [13], our platform additionally offers 65 tools (Figure 2) and online SVG editors, empowering researchers to freely adjust their plots for publication.

In the future, we intend to integrate EasyMicrobiome [23] and EasyMetagenome [29] pipelines in our platform, and constantly update the platform within half a year. Additionally, we will enhance the platform with English video tutorials. While the website is currently accessible through Google Translate, our future plans include updating the English version to enhance accessibility for international researchers.

## CONCLUSION

In summary, the Wekemo Bioincloud platform emerges as a valuable solution to the escalating challenges presented by the growing volume of meta‐omics data in microbial community studies. This specialized platform encompasses 22 workflows and 65 graphical tools, allowing for the modification of vector outputs. By addressing scalability issues inherent in existing bioinformatics tools, Wekemo Bioincloud enhances the platform's flexibility and user‐friendly experience.

## METHODS

The Wekemo Bioincloud is designed as a web application, employing Javascript, HTML, Vue, and Bootstrap for front‐end development. For back‐end data preprocessing and analysis, it incorporates various widely used ‐omics analysis software/tools. The steps include, but are not limited to, quality control, removal of host contamination, filtering rRNA reads and chimeras, addressing redundancy, binning or mapping, splicing or assembling, species or gene annotation, quantification of species, genes, metabolites, proteins, and functional prediction or annotation, as well as the analysis of diversity, differences, correlations, and phylogeny.

All detailed information on all software/tools for each workflow is available on our website. Here, we briefly outline the steps of metagenome workflow (reference‐based). In summary, Trimmomatic [7] is employed for quality control, read filtering, and base correction for FASTQ data. The remaining reads were aligned to the host genome reference by Bowtie [63] to remove host DNA contamination. Kraken [9] is utilized to sequence abundance, MetaPhlAn and mOTUs3 for taxonomic abundance [64], and HUMAnN [10] is employed to identify microbial functions. The output file with  .qzv file can be viewed using QIIME 2 [5], and most statistical analyses and plots are generated based on R scripts.

## AUTHOR CONTRIBUTIONS

Shunyao Jiang and Guoxing Zhang developed the platform. Yong‐Xin Liu and Shunyao Jiang conceived and coordinated the study. Yunyun Gao drafted the manuscript. Yong‐Xin Liu, Guoxing Zhang, and Shunyao Jiang revised the manuscript. All authors have read the final manuscript and approved it for publication.

## CONFLICT OF INTEREST STATEMENT

The authors declare no conflict of interest.

## Supporting information


**Table S1:** Overview of publications citing Wekemo Bioincloud on Google Scholar until December 6, 2023.

## Data Availability

Data sharing does not applicable to this article as no data sets were generated or analyzed during the current study. All demonstration data for visualization purposes are available on the Wekemo Bioincloud website (https://www.bioincloud.tech/). Supplementary materials (tables, scripts, graphical abstract, slides, videos, Chinese translated version and update materials) may be found in the online DOI or iMeta Science http://www.imeta.science/.
